# Association between anthropometric indices and hypertension: identifying optimal cutoff points for U.S. adults across different populations

**DOI:** 10.3389/fphar.2024.1503059

**Published:** 2024-12-12

**Authors:** Xueliang Zhang, Yan Nie, Dan Li, Chunhua Zhou

**Affiliations:** ^1^ Department of Pharmacy, The Second Hospital of Hebei Medical University, Shijiazhuang, Hebei, China; ^2^ Department of Pharmacy, The Third Hospital of Hebei Medical University, Shijiazhuang, Hebei, China; ^3^ Department of Clinical Pharmacy, The First Hospital of Hebei Medical University, Shijiazhuang, Hebei, China; ^4^ Department of the Technology Innovation Center for Artificial Intelligence in Clinical Pharmacy of Hebei Province, Shijiazhuang, China

**Keywords:** hypertension, anthropometric indices, cutoff points, hypertension prediction, stratified analysis

## Abstract

**Objective:**

This study compares the relationships between five anthropometric indices, a body shape index (ABSI), body roundness index (BRI), waist circumference (WC), body mass index (BMI) and waist-to-height ratio (WHtR), and hypertension, assessing their predictive capacities. The aim is to determine the specific numerical changes in hypertension incidence, systolic blood pressure (SBP) and diastolic blood pressure (DBP) for each increase in standard deviation of these indices, and to identify the optimal predictive indicators for different populations, including the calculation of cutoff values.

**Methods:**

This study used data from the NHANES datasets spanning 2007 to 2018. Logistic regression analysis was used to quantify the associations between these anthropometric indices and hypertension, calculating β coefficients and odds ratios (ORs). Receiver operating characteristic (ROC) analysis was used to evaluate the predictive ability of each index for hypertension.

**Results:**

For each increase in standard deviation in WC, BMI, WHtR, ABSI and BRI, the prevalence of hypertension increased by 33% (95% CI: 27%–40%), 32% (95% CI: 26%–38%), 35% (95% CI: 28%–42%), 9% (95% CI: 4%–16%) and 32% (95% CI: 26%–38%), respectively. The SBP correspondingly increased by 2.36 mmHg (95% CI: 2.16–2.56), 2.41 mmHg (95% CI: 2.21–2.60), 2.48 mmHg (95% CI: 2.28–2.68), 0.42 mmHg (95% CI: 0.19–0.66) and 2.46 mmHg (95% CI: 2.26–2.66), respectively. Similarly, DBP increased by 1.83 mmHg (95% CI: 1.68–1.98), 1.72 mmHg (95% CI: 1.58–1.87), 1.72 mmHg (95% CI: 1.57–1.88), 0.44 mmHg (95% CI: 0.27–0.62) and 1.64 mmHg (95% CI: 1.48–1.79). In the youth and middle-aged groups, WC had the best predictive ability, with AUCs of 0.749 and 0.603, respectively. Among the elderly group, the AUCs for all five indices ranged between 0.5 and 0.52.

**Conclusion:**

Increases in WC, BMI, WHtR and BRI are significantly associated with higher incidences of hypertension and increases in SBP and DBP, while the impact of ABSI on blood pressure is relatively weak. Stratified analysis indicates significant age-related differences in the predictive value of these indices, with the strongest associations observed in the youth group, followed by the middle age group, and the weakest in the elderly. WC demonstrates excellent predictive ability across youth populations.

## 1 Introduction

Hypertension is a widely recognized chronic disease and a significant threat to global health (Moraes-Silva et al., 2017). In recent years, its prevalence has increased steadily, driven by social changes, including changes in lifestyle and dietary habits (NCD Risk Factor Collaboration, 2021). Hypertension is a leading risk factor for stroke, cardiovascular disease and renal failure, and is among the primary causes of mortality worldwide. Cardiovascular-related deaths now exceed those attributed to any other reason, with more than three-quarters of these deaths occurring in low- and middle-income countries (World Health Organization, 2021). By 2025, the global prevalence of hypertension is projected to increase by 60%, affecting approximately 1.56 billion people (Kearney et al., 2005). If global hypertension control rates rise to 50%, it is estimated that between 2023 and 2050, up to 76 million deaths, 120 million strokes, 79 million heart attacks and 17 million cases of heart failure could be prevented (Pickersgill et al., 2022). In the United States, the burden of hypertension is particularly severe, with more than 100 million individuals expected to suffer from this condition (Fryar and Zhang, 2017; Dorans et al., 2018). Thus, efforts to prevent and control hypertension are urgently needed.

Obesity is one of the most significant modifiable risk factors contributing to hypertension, as confirmed by numerous studies (Kotsis et al., 2015; Ge et al., 2021). The American Heart Association (AHA) has emphasized the need to prioritize hypertension as a modifiable risk factor for cardiovascular disease mortality (Whelton et al., 2018). Due to their simplicity and cost-effectiveness, obesity-related indices have become the preferred tools for health risk assessment and screening (Zhang et al., 2024; Suárez et al., 2024). However, despite advances in hypertension pharmacotherapy and the fast-paced modern lifestyle, there is an over-reliance on medication, leading to a diminished emphasis on lifestyle modifications, particularly in obesity management, which is often considered challenging for patients.

Lifestyle modifications are consistently emphasized in hypertension treatment guidelines (Flack and Adekola, 2020). However, for clinical physicians and pharmacists, accurately assessing changes in patient body composition and identifying the most relevant anthropometric indices related to hypertension remain critical challenges. It is still debated whether these indices directly influence blood pressure and to what extent, with limited research providing direct evidence on the predictive value of these indices for hypertension (Kim et al., 2016; Kuciene and Dulskiene, 2019; Wu et al., 2022). If clinicians inform patients that reducing a specific anthropometric index correlates a measurable reduction in blood pressure, it could improve patient adherence to lifestyle interventions.

This study focuses on five non-invasive anthropometric indices: waist circumference (WC), body mass index (BMI), waist-to-height ratio (WHtR), a body shape index (ABSI) and body roundness index (BRI). Indices requiring lipid testing, such as the atherogenic index of plasma (AIP), lipid accumulation product (LAP), visceral adiposity index (VAI) and triglyceride-glucose index (TyG), were excluded due to their invasive nature (Li and Zeng, 2024). These five non-invasive indices are critical for evaluating overall health, and their associations with hypertension have gained increasing academic attention. Although existing research has examined the relationship between these indices and hypertension, the conclusions are inconsistent, and few studies have directly quantified the relationship between specific anthropometric indices and blood pressure (Cheung, 2014; Chang et al., 2016; Shen et al., 2017; Choi et al., 2018; Flack and Adekola, 2020; Maciel de Oliveira et al., 2022; Zhang et al., 2023; Li and Zeng, 2024; Tao et al., 2024). This is particularly true for newer indices, such as ABSI and BRI. Previous research has explored the relationship between anthropometric indices and hypertension in older Chinese populations, highlighting sex and age differences (Wang et al., 2018). This prompted the current study to conduct a stratified analysis by sex and age.

This study aims to conduct a detailed analysis of the relationship between these anthropometric indices and hypertension. This research seeks to provide clinical physicians and pharmacists with more accurate and scientific references to guide the prevention and treatment of hypertension, thereby reducing the incidence and complications of hypertension and safeguarding public health.

## 2 Materials and methods

### 2.1 The national health and nutrition examination survey (NHANES)

The National Health and Nutrition Examination Survey (NHANES) plays a crucial role in the field of public health. As a long-term, ongoing epidemiological survey, NHANES employs rigorous and comprehensive multi-stage probability sampling methods to select representative samples from the diverse U.S. populations. This robust design ensures both the breadth and accuracy of the research findings, offering valuable data to assess the health and nutritional status of adults and children in the United States (Li and Zeng, 2024; Liu et al., 2024).

The NHANES study protocol undergoes rigorous scientific review and careful planning, with full approval from the Institutional Review Board (IRB) of the National Center for Health Statistics (NCHS). All participants voluntarily sign informed consent forms after being fully informed of the study’s objectives, procedures and potential risks, ensuring the investigation’s legal and ethical integrity (www.cdc.gov/nchs/nhanes/irba98.htm).

### 2.2 Study design

**FIGURE 1:**
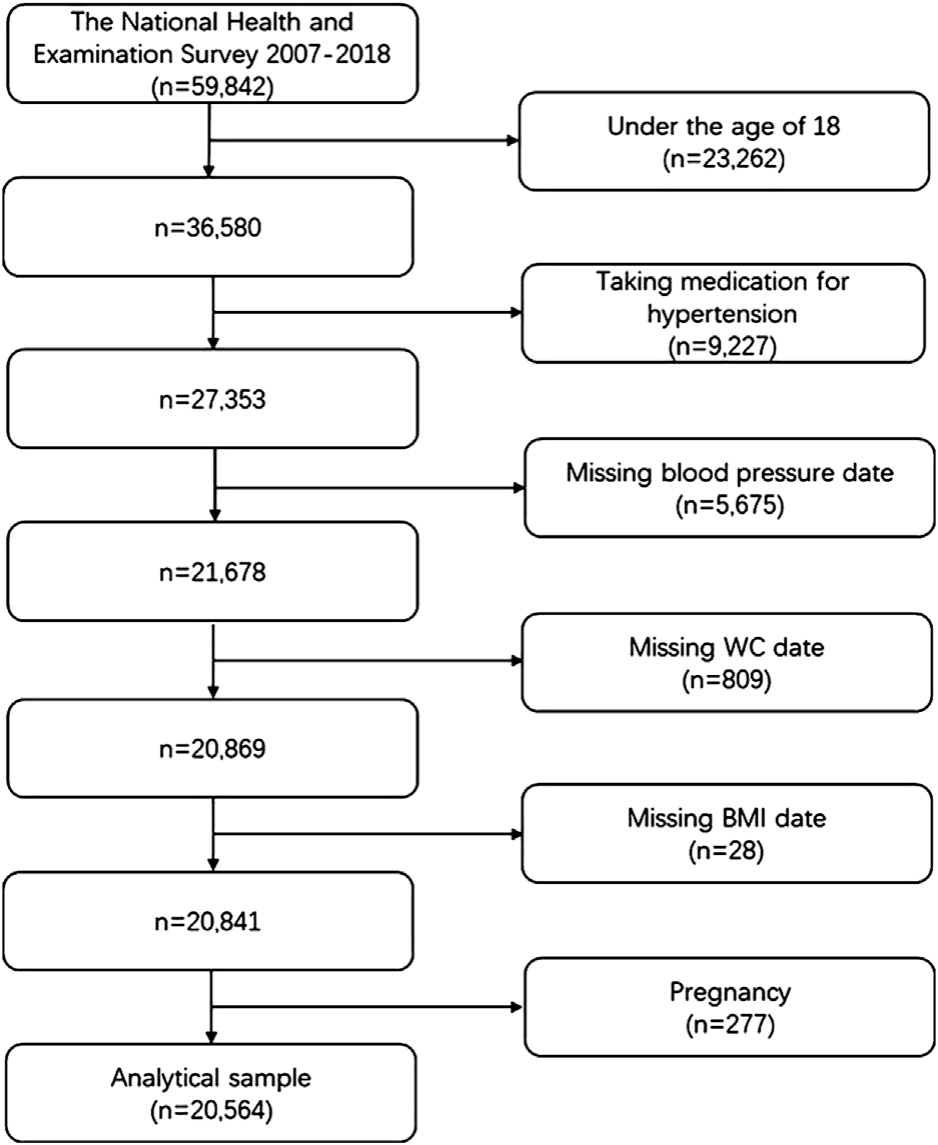
Flowchart of the study.

This study compiled data from six NHANES survey cycles, spanning 2007 to 2018, for 59,842 participants. After applying the exclusion criteria (Figure 1), individuals under 18 years of age, participants using antihypertensive medications, those with missing key data, and pregnant individuals were excluded. As a result, 20,564 samples were retained for analysis, with participants ranging in age from 18 to 80 years, the maximum age available in the NHANES database.

In this study, the WC and BMI were obtained directly from the NHANES database, while WHtR, ABSI and BRI were calculated using the following established formulas (Equations 1–3). Systolic and diastolic blood pressure values were determined by averaging three measurements in the database.

$$WHtR=\frac{\text{WC}}{\text{Height}}$$ (1)

$$ABSI=\frac{\text{WC}}{BMI^{\frac{2}{3}}{\text{Height}}^{\frac{1}{2}}}$$ (2)

$$BRI=364.2-365.5\times \sqrt{1-\frac{\text{WC}/{\left(2\pi \right)}^{2}}{{\left(0.5\;\text{Height}\right)}^{2}}}$$ (3)

The diagnostic criteria for hypertension in this study follow the standards outlined in the World Health Organization (WHO) 2021 guidelines for the pharmacological treatment of adult hypertension. According to these guidelines, systolic blood pressure (SBP) ≥140 mmHg and/or diastolic blood pressure (DBP) ≥90 mmHg are considered indicative of hypertension (World Health Organization, 2021).

Various potential confounding factors that could influence the outcomes were collected from the NHANES database. These factors included sex (male, female), age (18–80 years), race/ethnicity (Mexican American, Other Hispanic, Non-Hispanic White, Non-Hispanic Black, Other Race Including Multi-Racial), education level (less than 9th grade, 9th-11th grade [including 12th grade without a diploma], high school graduate/GED or equivalent, some college or AA degree, college graduate or above, missing) and monthly family income (ranging from $0-$799 to $8,400 and more, including missing data).

Other variables included average daily alcohol consumption in the past year (in cups) [1–4, 5–9, 10–15, 16 and over, missing], where one cup is defined as 12 ounces of beer, 5 ounces of wine, or 1.5 ounces of liquor. Marital status (married, widowed, divorced, separated, never married, living with partner, missing), average daily exercise time (minutes) [10–30, 31–60, 61–120, 121–240, 241 and over, missing], and average daily smoking quantity in the past 30 days (cigarettes) [0–10, 11–20, 21–30, 31–40, 41 and over, missing] were also collected.

For race/ethnicity, the following codes were used: Mexican American (race 1), Other Hispanic (race 2), Non-Hispanic White (race 3), Non-Hispanic Black (race 4) and Other Race Including Multi-Racial (race 5). Age groups were categorized as follows: youth (18–35 years), middle-aged (36–59 years) and elderly (60–80 years).

### 2.3 Statistical analysis

Statistical analyses were performed with EmpowerStats (version 2.0) and R (version 3.4.3). Continuous variables were presented as mean ± standard deviation (SD), and categorical variables were expressed as frequency (%). Baseline characteristics of the study population were described according to hypertension status. The Kruskal–Wallis rank sum test was employed for continuous variables, while Fisher’s exact test was applied for categorical variables with expected counts less than 10. The anthropometric indices were analyzed as continuous variables, using the standard deviation increment for each index.

Multiple logistic regression analysis was performed to calculate β coefficients and odds ratios (ORs). Receiver operating characteristic (ROC) curves were generated to assess the discriminatory ability of each anthropometric index to predict hypertension. Stratified analyses were conducted to identify the optimal predictive indicators across different genders, races and age groups. The cut-off values corresponding to the maximum Youden index were determined. Additionally, Bootstrap resampling with 500 iterations was used to evaluate the stability of the results in terms of the area under the ROC curve (AUC). Statistical significance was defined as *p* < 0.05.

## 3 Results

### 3.1 Characteristics of the study population

A total of 20,564 samples were included, categorized based on hypertension status (Table 1). Of these, 2,371 participants were diagnosed with hypertension. The hypertension group exhibited significantly higher mean values for age, WC, BMI, WHtR and BRI, compared to the non-hypertension group (*p* < 0.001). The males made up 58.96% of the hypertension group, of the hypertension group, significantly higher than the proportion of females (41.04%) (*p* < 0.001). However, no significant differences were observed between the groups regarding smoking and exercise habits (*p* > 0.05).

**TABLE 1:** Characteristics of the study population.

Hypertension	Negative	Positive	P*
Participants, n	18,193	2,371	
Age (years), Mean ± SD	40.59 ± 16.24	56.61 ± 15.18	<0.001
WC(cm), Mean ± SD	95.27 ± 15.81	101.58 ± 16.11	<0.001
BMI(kg/m^2^), Mean ± SD	27.94 ± 6.45	29.47 ± 7.05	<0.001
WHtR, Mean ± SD	0.57 ± 0.10	0.61 ± 0.09	<0.001
ABSI, Mean ± SD	0.08 ± 0.00	0.08 ± 0.00	<0.001
BRI, Mean ± SD	4.92 ± 2.18	5.78 ± 2.32	<0.001
Gender, n (%)			<0.001
Male	9,077 (49.89%)	1,398 (58.96%)	
Female	9,116 (50.11%)	973 (41.04%)	
Race, n (%)			<0.001
Mexican American	3,149 (17.31%)	352 (14.85%)	
Other Hispanic	2046 (11.25%)	246 (10.38%)	
Non-Hispanic White	7,145 (39.27%)	875 (36.90%)	
Non-Hispanic Black	3,267 (17.96%)	616 (25.98%)	
Other Race - Including Multi-Racial	2,586 (14.21%)	282 (11.89%)	
Education level, n (%)			<0.001
Less than 9th grade	1,460 (8.03%)	317 (13.37%)	
9–11th grade (Includes 12th grade with no diploma)	2,678 (14.72%)	374 (15.77%)	
High school graduate/GED or equivalent	4,502 (24.75%)	614 (25.90%)	
Some college or AA degree	5,172 (28.43%)	613 (25.85%)	
College graduate or above	4,371 (24.03%)	451 (19.02%)	
Missing	10 (0.05%)	2 (0.08%)	
Monthly family income, n (%)			<0.001
$0–$799	1,475 (8.11%)	204 (8.60%)	
$800–$1,649	2,999 (16.48%)	433 (18.26%)	
$1,650–$2,899	3,071 (16.88%)	447 (18.85%)	
$2,900–$4,599	3,161 (17.37%)	403 (17.00%)	
$4,600–$6,249	1874 (10.30%)	192 (8.10%)	
$6,250–$8,399	1,209 (6.65%)	114 (4.81%)	
$8,400 and over	2012 (11.06%)	220 (9.28%)	
Missing	2,392 (13.15%)	358 (15.10%)	
Average alcohol drinks/day—past 12 months, n (%)			<0.001
1–4	9,941 (54.64%)	1,203 (50.74%)	
5–9	1,619 (8.90%)	192 (8.10%)	
10–15	411 (2.26%)	62 (2.61%)	
16 and over	54 (0.30%)	5 (0.21%)	
Missing	6,168 (33.90%)	909 (38.34%)	
Marital status, n (%)			<0.001
Married	8,671 (47.66%)	945 (39.86%)	
Widowed	715 (3.93%)	86 (3.63%)	
Divorced	1,695 (9.32%)	205 (8.65%)	
Separated	595 (3.27%)	70 (2.95%)	
Never married	3,709 (20.39%)	576 (24.29%)	
Living with partner	1,676 (9.21%)	217 (9.15%)	
Missing	1,132 (6.22%)	272 (11.47%)	
Activities time (Minutes)/day, n (%)			0.293
10–30	1,065 (5.85%)	133 (5.61%)	
31–60	2,197 (12.08%)	303 (12.78%)	
61–120	1,342 (7.38%)	166 (7.00%)	
121–240	495 (2.72%)	61 (2.57%)	
241 and over	47 (0.26%)	12 (0.51%)	
Missing	13,047 (71.71%)	1,696 (71.53%)	
Cigarettes/day during past 30 days			0.424
0–10	2,481 (13.64%)	327 (13.79%)	
11–20	1,143 (6.28%)	152 (6.41%)	
21–30	186 (1.02%)	16 (0.67%)	
31–40	76 (0.42%)	15 (0.63%)	
41 and over	27 (0.15%)	3 (0.13%)	
Missing	14,280 (78.49%)	1,858 (78.36%)	

Results in the table: Mean + SD/N (%).

*p*-value*: For continuous variables, it is obtained using the Kruskal–Wallis rank-sum test; for count variables with theoretical numbers <10, it is obtained using Fisher’s exact probability test.

### 3.2 Univariate analysis

In the univariate analysis (Table 2), females had a hypertension prevalence rate that was 0.69 times that of males [95% confidence interval (CI): 0.64–0.76], using males as the reference group. Females also exhibited lower SBP and DBP by 5.77 mmHg (95% CI: 5.35–6.20) and 2.80 mmHg (95% CI: 2.50–3.10).

**TABLE 2:** Univariate analysis.

	Statistics	Hypertension (OR)	Mean systolic blood pressure (mmHg) (β)	Mean diastolic blood pressure (mmHg) (β)
Age (years)	42.44 ± 16.91	1.06 (1.06, 1.06) <0.0001	0.39 (0.38, 0.40) <0.0001	0.10 (0.09, 0.11) <0.0001
Gender				
Male	10,475 (50.94%)	1.0	0	0
Female	10,089 (49.06%)	0.69 (0.64, 0.76) <0.0001	−5.77 (−6.20, −5.35) <0.0001	−2.80 (−3.10, −2.50) <0.0001
Race				
Mexican American	3,501 (17.02%)	1.0	0	0
Other Hispanic	2,292 (11.15%)	1.08 (0.91, 1.28) 0.4065	−0.11 (−0.95, 0.72) 0.7882	0.16 (−0.41, 0.74) 0.5774
Non-Hispanic White	8,020 (39.00%)	1.10 (0.96, 1.25) 0.1708	0.42 (−0.21, 1.05) 0.1885	1.17 (0.73, 1.60) <0.0001
Non-Hispanic Black	3,883 (18.88%)	1.69 (1.47, 1.94) <0.0001	3.67 (2.95, 4.40) <0.0001	1.89 (1.39, 2.39) <0.0001
Other Race - Including Multi-Racial	2,868 (13.95%)	0.98 (0.83, 1.15) 0.7688	−1.41 (−2.19, −0.63) 0.0004	2.27 (1.73, 2.80) <0.0001
Education level				
Less than 9th grade	1,777 (8.64%)	1.0	0	0
9–11th grade (Include 12th grade with no diploma)	3,052 (14.84%)	0.64 (0.55, 0.76) <0.0001	−3.74 (−4.67, −2.81) <0.0001	−0.30 (−0.94, 0.34) 0.3598
High school graduate/GED or equivalent	5,116 (24.88%)	0.63 (0.54, 0.73) <0.0001	−4.08 (−4.94, −3.22) <0.0001	−0.02 (−0.61, 0.57) 0.9433
Some college or AA degree	5,785 (28.13%)	0.55 (0.47, 0.63) <0.0001	−4.61 (−5.45, −3.76) <0.0001	0.97 (0.39, 1.55) 0.0011
College graduate or above	4,822 (23.45%)	0.48 (0.41, 0.56) <0.0001	−6.26 (−7.13, −5.40) <0.0001	1.37 (0.78, 1.96) <0.0001
Missing	12 (0.06%)	0.92 (0.20, 4.22) 0.9158	−6.10 (−15.10, 2.90) 0.1842	0.44 (−5.75, 6.64) 0.8882
Monthly family income				
$0–$799	1,679 (8.16%)	1.0	0	0
$800–$1,649	3,432 (16.69%)	1.04 (0.87, 1.25) 0.6354	0.94 (0.01, 1.87) 0.0476	−0.69 (−1.33, −0.06) 0.0332
$1,650–$2,899	3,518 (17.11%)	1.05 (0.88, 1.26) 0.5712	1.14 (0.22, 2.07) 0.0155	−0.04 (−0.68, 0.59) 0.8921
$2,900–$4,599	3,564 (17.33%)	0.92 (0.77, 1.10) 0.3737	−0.05 (−0.97, 0.88) 0.9232	0.21 (−0.42, 0.85) 0.5090
$4,600–$6,249	2066 (10.05%)	0.74 (0.60, 0.91) 0.0048	−0.84 (−1.86, 0.18) 0.1082	−0.12 (−0.82, 0.59) 0.7485
$6,250–$8,399	1,323 (6.43%)	0.68 (0.54, 0.87) 0.0019	−0.80 (−1.95, 0.34) 0.1706	0.56 (−0.23, 1.35) 0.1645
$8,400 and over	2,232 (10.85%)	0.79 (0.65, 0.97) 0.0226	−1.86 (−2.86, −0.85) 0.0003	1.20 (0.51, 1.89) 0.0007
Missing	2,750 (13.37%)	1.08 (0.90, 1.30) 0.3998	1.03 (0.06, 1.99) 0.0374	−0.16 (−0.82, 0.51) 0.6427
Average alcohol drinks/day—past 12 months				
1–4	11,144 (54.19%)	1.0	0	0
5–9	1811 (8.81%)	0.98 (0.83, 1.15) 0.8057	1.71 (0.92, 2.50) <0.0001	0.77 (0.23, 1.31) 0.0053
10–15	473 (2.30%)	1.25 (0.95, 1.64) 0.1145	2.04 (0.57, 3.50) 0.0064	1.43 (0.43, 2.44) 0.0052
16 and over	59 (0.29%)	0.77 (0.31, 1.92) 0.5676	−1.33 (−5.40, 2.75) 0.5235	−0.61 (−3.40, 2.19) 0.6704
Missing	7,077 (34.41%)	1.22 (1.11, 1.33) <0.0001	0.86 (0.39, 1.34) 0.0004	−0.94 (−1.27, −0.62) <0.0001
Marital status				
Married	9,616 (46.76%)	1.0	0	0
Widowed	801 (3.90%)	1.10 (0.87, 1.39) 0.4079	0.06 (−1.08, 1.21) 0.9146	0.73 (−0.05, 1.51) 0.0666
Divorced	1900 (9.24%)	1.11 (0.95, 1.30) 0.2013	0.27 (−0.51, 1.05) 0.5005	0.20 (−0.34, 0.73) 0.4707
Separated	665 (3.23%)	1.08 (0.84, 1.40) 0.5590	−0.42 (−1.67, 0.82) 0.5055	−0.95 (−1.80, −0.09) 0.0295
Never married	4,285 (20.84%)	1.42 (1.28, 1.59) <0.0001	1.42 (0.85, 1.99) <0.0001	1.52 (1.13, 1.91) <0.0001
Living with partner	1893 (9.21%)	1.19 (1.02, 1.39) 0.0310	0.77 (−0.01, 1.56) 0.0527	−0.14 (−0.68, 0.39) 0.6031
Missing	1,404 (6.83%)	2.20 (1.90, 2.56) <0.0001	5.41 (4.52, 6.30) <0.0001	5.51 (4.90, 6.12) <0.0001
Activities time (Minutes)/day				
10–30	1,198 (5.83%)	1.0	0	0
31–60	2,500 (12.16%)	1.10 (0.89, 1.37) 0.3691	0.16 (−0.93, 1.26) 0.7677	−0.59 (−1.34, 0.16) 0.1244
61–120	1,508 (7.33%)	0.99 (0.78, 1.26) 0.9383	−0.36 (−1.57, 0.84) 0.5562	−1.35 (−2.17, −0.52) 0.0014
121–240	556 (2.70%)	0.99 (0.72, 1.36) 0.9353	−0.50 (−2.10, 1.10) 0.5372	−1.54 (−2.64, −0.45) 0.0057
241 and over	59 (0.29%)	2.04 (1.06, 3.95) 0.0334	1.86 (−2.30, 6.01) 0.3810	−2.04 (−4.88, 0.81) 0.1604
Missing	14,743 (71.69%)	1.04 (0.86, 1.26) 0.6747	−2.52 (−3.45, −1.58) <0.0001	−3.20 (−3.84, −2.56) <0.0001
Cigarettes/day during past 30 days				
0–10	2,808 (13.65%)	1.0	0	0
11–20	1,295 (6.30%)	1.01 (0.82, 1.24) 0.9319	−0.34 (−1.39, 0.71) 0.5259	−0.59 (−1.31, 0.12) 0.1040
21–30	202 (0.98%)	0.65 (0.39, 1.10) 0.1099	−1.51 (−3.79, 0.76) 0.1911	−0.70 (−2.26, 0.85) 0.3755
31–40	91 (0.44%)	1.50 (0.85, 2.64) 0.1618	3.06 (−0.26, 6.38) 0.0706	0.01 (−2.26, 2.28) 0.9949
41 and over	30 (0.15%)	0.84 (0.25, 2.79) 0.7800	1.32 (−4.41, 7.04) 0.6523	−0.95 (−4.87, 2.96) 0.6339
Missing	16,138 (78.48%)	0.99 (0.87, 1.12) 0.8397	−2.45 (−3.09, −1.82) <0.0001	−2.73 (−3.16, −2.29) <0.0001
WC (cm) Z score	−0.00 ± 1.00	1.44 (1.39, 1.50) <0.0001	3.95 (3.74, 4.16) <0.0001	2.15 (2.00, 2.30) <0.0001
BMI (kg/m^2^) Z score	−0.00 ± 1.00	1.24 (1.19, 1.29) <0.0001	2.74 (2.52, 2.95) <0.0001	1.64 (1.50, 1.79) <0.0001
WHtR Z score	−0.00 ± 1.00	1.45 (1.40, 1.51) <0.0001	3.60 (3.39, 3.81) <0.0001	1.61 (1.46, 1.76) <0.0001
ABSI Z score	−0.00 ± 1.00	1.73 (1.66, 1.81) <0.0001	3.66 (3.45, 3.87) <0.0001	1.11 (0.96, 1.26) <0.0001
BRI Z score	−0.00 ± 1.00	1.40 (1.35, 1.45) <0.0001	3.45 (3.24, 3.66) <0.0001	1.52 (1.37, 1.66) <0.0001

Data in the table: β (95%CI) *p*-value/OR (95%CI) *p*-value.

Outcome variables: Hypertension; Mean systolic blood pressure (mmHg); Mean diastolic blood pressure (mmHg).

Exposure variables: Age (years); Gender; Race; Education level; Monthly family income; Avg alcohol drinks/day—past 12 months; Marital status; Activities time (Minutes)/day; Cigarettes/day during past 30 days; Waist (cm) Z score; ABSI Z score; BMI (kg/m2) Z score; WHtR Z score; BRI Z score.

Adjustment variables: None.

Regarding ethnicity, compared to race 1 (Mexican American) as the reference, race 3 (Non-Hispanic White) had a DBP of 1.17 mmHg higher (95% CI: 0.73–1.60). Race 4 (Non-Hispanic Black) showed a significantly higher hypertension prevalence rate, 1.69 times (95% CI: 1.47–1.94), with SBP 3.67 mmHg higher (95% CI: 2.95–4.40) and DBP1.89 mmHg higher (95% CI: 1.39–2.39) than race 1. Race 5 (Other Race Including Multi-Racial) exhibited a SBP that was 1.41 mmHg lower (95% CI: 0.63–2.19) and DBP 2.27 mmHg higher (95% CI: 1.73–2.80) than race 1. No statistically significant differences were observed for other factors.

This study also showed that alcohol consumption was associated with increased systolic and diastolic blood pressure. Additionally, higher levels of education and family income were associated with a decreasing trend in the prevalence of hypertension. Regarding marital status, individuals who were never married had a 1.42 times higher prevalence of hypertension (95% CI: 1.28–1.59) compared to those who were married, with systolic and diastolic pressures elevated by 1.42 mmHg (95% CI: 0.85–1.99) and 1.52 mmHg (95% CI: 1.13–1.91), respectively. Physical activity was associated with a reduction in diastolic pressure, while smoking did not show a statistically significant association with hypertension (*p* > 0.05). Without adjusting for confounders, each increase in standard deviation in the five body measurement indices was significantly associated with an increased incidence of hypertension and higher systolic and diastolic pressures (*p* < 0.0001).

### 3.3 Trend analysis

To validate the stability of these relationships, trend analyses were conducted by categorizing the five anthropometric indices into quartiles and transforming continuous variables into categorical variables (Table 3). Model I was unadjusted, and Model II was adjusted for gender, age and race. In contrast, Model III was further adjusted for gender, age, race, education level, family income, marital status, smoking status, alcohol consumption and physical activity. The trend was statistically significant (*p* < 0.01), indicating a clear association. However, among these variables, ABSI exhibited the weakest trend.

**TABLE 3:** Trend analysis.

Exposure	Model Ⅰ	Model Ⅱ	Model Ⅲ
Mean systolic blood pressure (mmHg) (β)			
WC (cm)			
Q1	0	0	0
Q2	4.73 (4.13, 5.33) <0.0001	1.56 (1.01, 2.11) <0.0001	1.60 (1.05, 2.14) <0.0001
Q3	7.87 (7.27, 8.47) <0.0001	3.04 (2.48, 3.61) <0.0001	2.96 (2.40, 3.52) <0.0001
Q4	10.38 (9.79, 10.98) <0.0001	5.81 (5.25, 6.37) <0.0001	5.54 (4.98, 6.11) <0.0001
P for trend	*p* < 0.01	*p* < 0.01	*p* < 0.01
BMI (kg/m^2^)			
Q1	0	0	0
Q2	3.56 (2.95, 4.17) <0.0001	1.19 (0.64, 1.74) <0.0001	1.29 (0.75, 1.83) <0.0001
Q3	5.72 (5.11, 6.33) <0.0001	2.63 (2.08, 3.19) <0.0001	2.58 (2.03, 3.14) <0.0001
Q4	7.38 (6.77, 7.99) <0.0001	5.87 (5.32, 6.43) <0.0001	5.69 (5.14, 6.24) <0.0001
P for trend	*p* < 0.01	*p* < 0.01	*p* < 0.01
WHtR			
Q1	0	0	0
Q2	4.03 (3.40, 4.66) <0.0001	1.30 (0.72, 1.87) <0.0001	1.45 (0.88, 2.02) <0.0001
Q3	7.25 (6.64, 7.86) <0.0001	3.01 (2.44, 3.59) <0.0001	2.89 (2.32, 3.46) <0.0001
Q4	9.84 (9.23, 10.45) <0.0001	6.29 (5.71, 6.87) <0.0001	5.96 (5.37, 6.54) <0.0001
P for trend	*p* < 0.01	*p* < 0.01	*p* < 0.01
ABSI			
Q1	0	0	0
Q2	2.42 (1.80, 3.04) <0.0001	0.58 (0.00, 1.16) 0.0490	0.49 (−0.08, 1.07) 0.0900
Q3	5.56 (4.92, 6.21) <0.0001	1.30 (0.69, 1.92) <0.0001	1.06 (0.44, 1.67) 0.0008
Q4	9.56 (8.92, 10.19) <0.0001	1.71 (1.05, 2.37) <0.0001	1.17 (0.52, 1.83) 0.0005
P for trend	*p* < 0.01	*p* < 0.01	*p* < 0.01
BRI			
Q1	0	0	0
Q2	3.97 (3.37, 4.57) <0.0001	1.13 (0.58, 1.68) <0.0001	1.24 (0.69, 1.79) <0.0001
Q3	7.39 (6.79, 7.99) <0.0001	3.33 (2.77, 3.90) <0.0001	3.17 (2.61, 3.74) <0.0001
Q4	9.59 (8.99, 10.19) <0.0001	6.32 (5.75, 6.89) <0.0001	5.97 (5.40, 6.54) <0.0001
P for trend	*p* < 0.01	*p* < 0.01	*p* < 0.01
Mean diastolic blood pressure(mmHg) (β)			
WC (cm)			
Q1	0	0	0
Q2	2.50 (2.08, 2.91) <0.0001	2.02 (1.60, 2.44) <0.0001	1.91 (1.49, 2.32) <0.0001
Q3	4.52 (4.11, 4.94) <0.0001	3.79 (3.36, 4.22) <0.0001	3.64 (3.21, 4.06) <0.0001
Q4	5.52 (5.11, 5.94) <0.0001	4.92 (4.49, 5.34) <0.0001	4.64 (4.21, 5.06) <0.0001
P for trend	*p* < 0.01	*p* < 0.01	*p* < 0.01
BMI (kg/m^2^)			
Q1	0	0	0
Q2	1.59 (1.17, 2.01) <0.0001	1.14 (0.72, 1.55) <0.0001	1.12 (0.71, 1.53) <0.0001
Q3	3.31 (2.89, 3.72) <0.0001	2.90 (2.48, 3.32) <0.0001	2.82 (2.41, 3.24) <0.0001
Q4	4.44 (4.02, 4.86) <0.0001	4.64 (4.22, 5.06) <0.0001	4.49 (4.07, 4.90) <0.0001
P for trend	*p* < 0.01	*p* < 0.01	*p* < 0.01
WHtR			
Q1	0	0	0
Q2	2.71 (2.28, 3.15) <0.0001	2.40 (1.96, 2.83) <0.0001	2.33 (1.89, 2.76) <0.0001
Q3	4.02 (3.60, 4.44) <0.0001	3.66 (3.22, 4.09) <0.0001	3.66 (3.22, 4.09) <0.0001
Q4	4.66 (4.24, 5.09) <0.0001	4.83 (4.39, 5.27) <0.0001	4.84 (4.40, 5.28) <0.0001
P for trend	*p* < 0.01	*p* < 0.01	*p* < 0.01
ABSI			
Q1	0	0	0
Q2	2.68 (2.25, 3.12) <0.0001	2.41 (1.98, 2.85) <0.0001	2.24 (1.81, 2.67) <0.0001
Q3	3.96 (3.51, 4.41) <0.0001	3.16 (2.70, 3.63) <0.0001	2.98 (2.51, 3.44) <0.0001
Q4	3.64 (3.20, 4.08) <0.0001	2.03 (1.53, 2.52) <0.0001	1.98 (1.48, 2.47) <0.0001
P for trend	*p* < 0.01	*p* < 0.01	*p* < 0.01
BRI			
Q1	0	0	0
Q2	2.60 (2.18, 3.02) <0.0001	2.25 (1.83, 2.66) <0.0001	2.23 (1.82, 2.64) <0.0001
Q3	4.04 (3.62, 4.46) <0.0001	3.71 (3.28, 4.14) <0.0001	3.72 (3.30, 4.15) <0.0001
Q4	4.41 (3.99, 4.83) <0.0001	4.61 (4.18, 5.05) <0.0001	4.65 (4.22, 5.08) <0.0001
P for trend	*p* < 0.01	*p* < 0.01	*p* < 0.01
Hypertension (OR)			
WC (cm)			
Q1	1.0	1.0	1.0
Q2	1.87 (1.62, 2.17) <0.0001	1.29 (1.10, 1.51) 0.0015	1.31 (1.12, 1.54) 0.0008
Q3	2.55 (2.21, 2.94) <0.0001	1.51 (1.30, 1.76) <0.0001	1.52 (1.31, 1.78) <0.0001
Q4	3.22 (2.80, 3.69) <0.0001	2.02 (1.74, 2.35) <0.0001	2.00 (1.72, 2.33) <0.0001
P for trend	*p* < 0.01	*p* < 0.01	*p* < 0.01
BMI (kg/m^2^)			
Q1	1.0	1.0	1.0
Q2	1.46 (1.28, 1.67) <0.0001	1.20 (1.04, 1.38) 0.0144	1.23 (1.06, 1.42) 0.0052
Q3	1.68 (1.48, 1.92) <0.0001	1.33 (1.16, 1.54) <0.0001	1.35 (1.17, 1.56) <0.0001
Q4	1.93 (1.70, 2.19) <0.0001	1.92 (1.67, 2.20) <0.0001	1.93 (1.68, 2.23) <0.0001
P for trend	*p* < 0.01	*p* < 0.01	*p* < 0.01
WHtR			
Q1	1.0	1.0	1.0
Q2	1.96 (1.67, 2.31) <0.0001	1.34 (1.13, 1.59) 0.0007	1.39 (1.17, 1.65) 0.0002
Q3	2.81 (2.42, 3.28) <0.0001	1.62 (1.38, 1.91) <0.0001	1.63 (1.38, 1.93) <0.0001
Q4	3.62 (3.11, 4.20) <0.0001	2.23 (1.90, 2.62) <0.0001	2.20 (1.87, 2.60) <0.0001
P for trend	*p* < 0.01	*p* < 0.01	*p* < 0.01
ABSI			
Q1	1.0	1.0	1.0
Q2	1.94 (1.63, 2.32) <0.0001	1.51 (1.26, 1.81) <0.0001	1.47 (1.22, 1.77) <0.0001
Q3	3.13 (2.64, 3.71) <0.0001	1.75 (1.46, 2.10) <0.0001	1.68 (1.40, 2.02) <0.0001
Q4	4.93 (4.18, 5.81) <0.0001	1.74 (1.44, 2.09) <0.0001	1.61 (1.33, 1.95) <0.0001
P for trend	*p* < 0.01	*p* < 0.01	*p* < 0.01
BRI			
Q1	1.0	1.0	1.0
Q2	1.91 (1.64, 2.22) <0.0001	1.31 (1.11, 1.53) 0.0010	1.34 (1.14, 1.57) 0.0004
Q3	2.70 (2.34, 3.12) <0.0001	1.61 (1.38, 1.88) <0.0001	1.60 (1.37, 1.88) <0.0001
Q4	3.40 (2.96, 3.91) <0.0001	2.19 (1.88, 2.56) <0.0001	2.16 (1.85, 2.52) <0.0001
P for trend	*p* < 0.01	*p* < 0.01	*p* < 0.01

Data in the table: β (95%CI) *p*-value/OR (95%CI) *p*-value.

Outcome variables: Hypertension; Mean systolic blood pressure (mmHg); Mean diastolic blood pressure (mmHg).

Exposure variables: Waist (cm) quartiles; BMI (kg/m^2^) quartiles; WHtR quartiles; ABSI, quartiles; BRI, quartiles.

Model I adjust for: None.

Model II, adjust for: Gender; Age (years); Race.

Model III, adjust for: Gender; Age (years); Race; Education level; Monthly family income; Avg alcohol drinks/day—past 12 months; Marital status; Activities time (Minutes)/day; Cigarettes/day during past 30 days.

### 3.4 Stratified analysis

A gender-stratified analysis (Table 4) revealed that ABSI had no significant impact on the occurrence of hypertension or DBP in females, while the remaining four anthropometric indices maintained stable associations. Among males, all five anthropometric indices exhibited consistent associations.

**TABLE 4:** Stratified analysis by gender.

Exposure	Gender = male	Gender = female	Total
Hypertension (OR)			
WC (cm) Z score	1.40 (1.32, 1.50) <0.0001	1.30 (1.21, 1.40) <0.0001	1.33 (1.27, 1.40) <0.0001
BM I (kg/m^2^) Z score	1.41 (1.32, 1.51) <0.0001	1.26 (1.18, 1.35) <0.0001	1.32 (1.26, 1.38) <0.0001
WHtR Z score	1.43 (1.34, 1.53) <0.0001	1.29 (1.20, 1.39) <0.0001	1.35 (1.28, 1.41) <0.0001
ABSI Z score	1.20 (1.11, 1.31) <0.0001	1.06 (0.98, 1.15) 0.1200	1.09 (1.04, 1.16) 0.0014
BRI Z score	1.41 (1.32, 1.51) <0.0001	1.26 (1.18, 1.35) <0.0001	1.32 (1.26, 1.38) <0.0001
Mean systolic blood pressure(mmHg) (β)			
WC (cm) Z score	2.32 (2.03, 2.62) <0.0001	2.56 (2.29, 2.83) <0.0001	2.36 (2.16, 2.56) <0.0001
BMI (kg/m^2^) Z score	2.45 (2.14, 2.75) <0.0001	2.35 (2.10, 2.59) <0.0001	2.41 (2.21, 2.60) <0.0001
WHtR Z score	2.48 (2.17, 2.79) <0.0001	2.55 (2.28, 2.81) <0.0001	2.48 (2.28, 2.68) <0.0001
ABSI Z score	1.05 (0.69, 1.42) <0.0001	0.48 (0.18, 0.78) 0.0019	0.42 (0.19, 0.66) 0.0003
BRI Z score	2.54 (2.23, 2.86) <0.0001	2.45 (2.19, 2.70) <0.0001	2.46 (2.26, 2.66) <0.0001
Mean diastolic blood pressure(mmHg) (β)			
WC (cm) Z score	2.20 (1.97, 2.43) <0.0001	1.51 (1.31, 1.70) <0.0001	1.83 (1.68, 1.98) <0.0001
BMI (kg/m^2^) Z score	2.27 (2.03, 2.51) <0.0001	1.33 (1.15, 1.51) <0.0001	1.72 (1.58, 1.87) <0.0001
WHtR Z score	2.26 (2.02, 2.51) <0.0001	1.33 (1.14, 1.52) <0.0001	1.72 (1.57, 1.88) <0.0001
ABSI Z score	0.82 (0.53, 1.11) <0.0001	0.21 (−0.01, 0.43) 0.0627	0.44 (0.27, 0.62) <0.0001
BRI Z score	2.23 (1.98, 2.48) <0.0001	1.24 (1.05, 1.43) <0.0001	1.64 (1.48, 1.79) <0.0001

Data in the table: β (95%CI) *p*-value/OR (95%CI) *p*-value.

Outcome variables: Hypertension; Mean systolic blood pressure (mmHg); Mean diastolic blood pressure (mmHg).

Exposure variables: ABSI Z score; Waist (cm) Z score; BMI (kg/m^2^) Z score; WHtR Z score; BRI Z score.

Model adjusted for: Race; Age; Education level; Monthly family income; Average alcohol drinks/day—past 12 months; Marital status; Activities time (Minutes)/day; Cigarettes/day during past 30 days.

When stratified by race (Table 5), ABSI had no significant impact on the occurrence of hypertension or SBP in races 1, 2, 3 and 5. Similarly, ABSI did not significantly impact DBP in races 1, 2 and 5. In contrast, the other four anthropometric indices demonstrated stable associations between all racial groups.

**TABLE 5:** Stratified analysis by race.

Exposure	Race = Mexican American	Race = other Hispanic	Race = non-Hispanic White	Race = non-Hispanic Black	Race = other race—including multi-racial	Total
Hypertension (OR)						
WC (cm) Z score	1.50 (1.31, 1.73) <0.0001	1.35 (1.13, 1.61) 0.0009	1.21 (1.12, 1.31) <0.0001	1.37 (1.25, 1.50) <0.0001	1.56 (1.34, 1.83) <0.0001	1.33 (1.27, 1.40) <0.0001
BMI (kg/m^2^) Z score	1.42 (1.25, 1.62) <0.0001	1.34 (1.14, 1.58) 0.0005	1.21 (1.12, 1.30) <0.0001	1.33 (1.23, 1.45) <0.0001	1.65 (1.41, 1.92) <0.0001	1.32 (1.26, 1.38) <0.0001
WHtR Z score	1.47 (1.28, 1.69) <0.0001	1.40 (1.17, 1.67) 0.0002	1.23 (1.13, 1.33) <0.0001	1.37 (1.26, 1.50) <0.0001	1.62 (1.38, 1.90) <0.0001	1.35 (1.28, 1.41) <0.0001
ABSI Z score	1.09 (0.94, 1.28) 0.2597	1.04 (0.87, 1.25) 0.6687	1.09 (1.00, 1.19) 0.0531	1.22 (1.09, 1.36) 0.0004	0.96 (0.81, 1.13) 0.6220	1.09 (1.04, 1.16) 0.0014
BRI Z score	1.39 (1.23, 1.58) <0.0001	1.35 (1.15, 1.59) 0.0004	1.21 (1.12, 1.30) <0.0001	1.36 (1.25, 1.48) <0.0001	1.59 (1.36, 1.86) <0.0001	1.32 (1.26, 1.38) <0.0001
Mean systolic blood pressure(mmHg) (β)						
WC (cm) Z score	2.49 (2.00, 2.99) <0.0001	2.62 (1.95, 3.29) <0.0001	2.18 (1.87, 2.49) <0.0001	2.35 (1.90, 2.80) <0.0001	2.82 (2.23, 3.41) <0.0001	2.36 (2.16, 2.56) <0.0001
BMI (kg/m^2^) Z score	2.39 (1.92, 2.86) <0.0001	2.65 (2.02, 3.28) <0.0001	2.28 (1.98, 2.59) <0.0001	2.21 (1.79, 2.64) <0.0001	3.15 (2.57, 3.74) <0.0001	2.41 (2.21, 2.60) <0.0001
WHtR Z score	2.57 (2.08, 3.07) <0.0001	2.76 (2.09, 3.42) <0.0001	2.34 (2.02, 2.65) <0.0001	2.38 (1.93, 2.84) <0.0001	3.04 (2.44, 3.64) <0.0001	2.48 (2.28, 2.68) <0.0001
ABSI Z score	0.45 (−0.12, 1.03) 0.1219	0.19 (−0.52, 0.90) 0.6057	0.40 (0.03, 0.76) 0.0321	1.11 (0.56, 1.67) <0.0001	−0.12 (−0.72, 0.49) 0.7030	0.42 (0.19, 0.66) 0.0003
BRI Z score	2.46 (1.99, 2.93) <0.0001	2.72 (2.06, 3.37) <0.0001	2.31 (2.00, 2.62) <0.0001	2.42 (1.97, 2.87) <0.0001	3.15 (2.52, 3.78) <0.0001	2.46 (2.26, 2.66) <0.0001
Mean diastolic blood pressure(mmHg) (β)						
WC (cm) Z score	1.88 (1.50, 2.25) <0.0001	1.84 (1.35, 2.33) <0.0001	1.84 (1.60, 2.07) <0.0001	1.69 (1.35, 2.02) <0.0001	1.89 (1.44, 2.33) <0.0001	1.83 (1.68, 1.98) <0.0001
BMI (kg/m^2^) Z score	1.66 (1.31, 2.02) <0.0001	1.74 (1.28, 2.21) <0.0001	1.76 (1.53, 1.99) <0.0001	1.49 (1.17, 1.82) <0.0001	2.01 (1.56, 2.45) <0.0001	1.72 (1.58, 1.87) <0.0001
WHtR Z score	1.67 (1.29, 2.05) <0.0001	1.60 (1.11, 2.08) <0.0001	1.74 (1.50, 1.98) <0.0001	1.65 (1.31, 1.99) <0.0001	1.90 (1.44, 2.36) <0.0001	1.72 (1.57, 1.88) <0.0001
ABSI Z score	0.31 (−0.13, 0.75) 0.1656	−0.18 (−0.70, 0.33) 0.4856	0.44 (0.17, 0.72) 0.0015	1.05 (0.64, 1.47) <0.0001	0.03 (−0.43, 0.49) 0.8957	0.44 (0.27, 0.62) <0.0001
BRI Z score	1.47 (1.11, 1.83) <0.0001	1.51 (1.03, 1.98) <0.0001	1.64 (1.40, 1.87) <0.0001	1.63 (1.29, 1.97) <0.0001	1.92 (1.44, 2.40) <0.0001	1.64 (1.48, 1.79) <0.0001

Data in the table: β (95%CI) *p*-value/OR (95%CI) p-value.

Outcome variables: Hypertension; Mean systolic blood pressure (mmHg); Mean diastolic blood pressure (mmHg).

Exposure variables: ABSI Z score; Waist (cm) Z score; BMI (kg/m^2^) Z score; WHtR Z score; BRI Z score.

Model adjusted for: Gender; Age; Education level; Monthly family income; Average alcohol drinks/day—past 12 months; Marital status; Activities time (Minutes)/day; Cigarettes/day during past 30 days.

In the age-stratified analysis (Table 6), only ABSI significantly affected SBP in the elderly group, with an increase of 1.03 mmHg (95% CI: 0.36–1.69) per standard deviation. The other indices either had non-significant effects or exhibited opposite trends, suggesting that these anthropometric indices had limited predictive value for blood pressure in older people. However, the results were significant and consistent in the younger and middle-aged groups.

**TABLE 6:** Stratified analysis by age.

Exposure	Age (years) = <36	Age (years) = ≥36, <60	Age (years) = ≥60	Total
Hypertension (OR)				
WC (cm) Z score	2.11 (1.89, 2.35) <0.0001	1.40 (1.31, 1.50) <0.0001	0.96 (0.88, 1.05) 0.3618	1.33 (1.27, 1.40) <0.0001
BMI (kg/m^2^) Z score	1.93 (1.75, 2.13) <0.0001	1.31 (1.23, 1.40) <0.0001	0.95 (0.87, 1.04) 0.2495	1.29 (1.23, 1.35) <0.0001
WHtR Z score	2.16 (1.93, 2.42) <0.0001	1.42 (1.33, 1.52) <0.0001	1.00 (0.92, 1.08) 0.9309	1.36 (1.29, 1.42) <0.0001
ABSI Z score	1.64 (1.39, 1.93) <0.0001	1.32 (1.22, 1.43) <0.0001	1.08 (1.00, 1.17) 0.0639	1.21 (1.14, 1.27) <0.0001
BRI Z score	2.00 (1.80, 2.22) <0.0001	1.37 (1.29, 1.46) <0.0001	1.00 (0.92, 1.08) 0.9367	1.33 (1.27, 1.39) <0.0001
Mean systolic blood pressure(mmHg) (β)				
WC (cm) Z score	3.15 (2.95, 3.35) <0.0001	3.23 (2.89, 3.57) <0.0001	0.33 (−0.38, 1.03) 0.3616	2.70 (2.50, 2.90) <0.0001
BMI (kg/m^2^) Z score	3.06 (2.86, 3.26) <0.0001	2.88 (2.55, 3.21) <0.0001	0.14 (−0.57, 0.85) 0.7073	2.59 (2.39, 2.79) <0.0001
WHtR Z score	3.15 (2.95, 3.36) <0.0001	3.36 (3.01, 3.70) <0.0001	0.65 (−0.05, 1.35) 0.0688	2.84 (2.64, 3.04) <0.0001
ABSI Z score	1.12 (0.86, 1.38) <0.0001	1.58 (1.20, 1.97) <0.0001	1.03 (0.36, 1.69) 0.0025	1.11 (0.89, 1.34) <0.0001
BRI Z score	3.19 (2.98, 3.40) <0.0001	3.18 (2.85, 3.52) <0.0001	0.63 (−0.06, 1.31) 0.0717	2.78 (2.58, 2.98) <0.0001
Mean diastolic blood pressure(mmHg) (β)				
WC (cm) Z score	2.15 (1.94, 2.35) <0.0001	1.70 (1.47, 1.93) <0.0001	−0.03 (−0.45, 0.39) 0.8915	1.66 (1.51, 1.80) <0.0001
BMI (kg/m^2^) Z score	1.87 (1.66, 2.07) <0.0001	1.55 (1.33, 1.77) <0.0001	0.27 (−0.15, 0.70) 0.2086	1.51 (1.37, 1.66) <0.0001
WHtR Z score	2.12 (1.91, 2.32) <0.0001	1.63 (1.40, 1.86) <0.0001	−0.25 (−0.67, 0.17) 0.2444	1.58 (1.44, 1.73) <0.0001
ABSI Z score	1.61 (1.36, 1.86) <0.0001	0.50 (0.25, 0.76) 0.0001	−1.02 (−1.41, −0.62) <0.0001	0.66 (0.50, 0.83) <0.0001
BRI Z score	2.12 (1.91, 2.33) <0.0001	1.51 (1.28, 1.73) <0.0001	−0.25 (−0.66, 0.16) 0.2296	1.51 (1.37, 1.66) <0.0001

Data in the table: β (95%CI) *p*-value/OR (95%CI) *p*-value.

Outcome variables: Hypertension; Mean systolic blood pressure (mmHg); Mean diastolic blood pressure (mmHg).

Exposure variables: ABSI Z score; Waist (cm) Z score; BMI (kg/m^2^) Z score; WHtR Z score; BRI Z score.

Model adjusted for: Race; Gender; Education level; Monthly family income; Average alcohol drinks/day—past 12 months; Marital status; Activities time (Minutes)/day; Cigarettes/day during past 30 days.

These stratified analyses indicate that ABSI has a weaker relationship with hypertension, with its influence significantly moderated by gender, race and age.

### 3.5 Multivariate regression analysis

Multivariate regression analysis was conducted to assess the impact of each anthropometric index on the occurrence of hypertension, SBP and DBP (Table 7). Model I was unadjusted, and Model II was adjusted for gender, age and race. In contrast, Model III was further adjusted for gender, age, race, education level, family income, marital status, smoking status, alcohol consumption and physical activity.

**TABLE 7:** Multivariate regression analysis.

Exposure	Model Ⅰ	Model Ⅱ	Model Ⅲ
Hypertension (OR)			
WC (cm) Z score	1.44 (1.39, 1.50) <0.0001	1.34 (1.28, 1.41) <0.0001	1.33 (1.27, 1.40) <0.0001
BMI (kg/m^2^) Z score	1.24 (1.19, 1.29) <0.0001	1.33 (1.27, 1.39) <0.0001	1.32 (1.26, 1.38) <0.0001
WHtR Z score	1.45 (1.40, 1.51) <0.0001	1.37 (1.30, 1.43) <0.0001	1.35 (1.28, 1.41) <0.0001
ABSI Z score	1.73 (1.66, 1.81) <0.0001	1.13 (1.07, 1.19) <0.0001	1.09 (1.04, 1.16) 0.0014
BRI Z score	1.40 (1.35, 1.45) <0.0001	1.34 (1.28, 1.40) <0.0001	1.32 (1.26, 1.38) <0.0001
Mean systolic blood pressure(mmHg) (β)			
WC (cm) Z score	3.95 (3.74, 4.16) <0.0001	2.47 (2.27, 2.67) <0.0001	2.36 (2.16, 2.56) <0.0001
BMI (kg/m^2^) Z score	2.74 (2.52, 2.95) <0.0001	2.50 (2.31, 2.70) <0.0001	2.41 (2.21, 2.60) <0.0001
WHtR Z score	3.60 (3.39, 3.81) <0.0001	2.63 (2.43, 2.83) <0.0001	2.48 (2.28, 2.68) <0.0001
ABSI Z score	3.66 (3.45, 3.87) <0.0001	0.65 (0.42, 0.88) <0.0001	0.42 (0.19, 0.66) 0.0003
BRI Z score	3.45 (3.24, 3.66) <0.0001	2.62 (2.42, 2.82) <0.0001	2.46 (2.26, 2.66) <0.0001
Mean diastolic blood pressure(mmHg) (β)			
WC (cm) Z score	2.15 (2.00, 2.30) <0.0001	1.93 (1.78, 2.08) <0.0001	1.83 (1.68, 1.98) <0.0001
BMI (kg/m^2^) Z score	1.64 (1.50, 1.79) <0.0001	1.79 (1.65, 1.94) <0.0001	1.72 (1.58, 1.87) <0.0001
WHtR Z score	1.61 (1.46, 1.76) <0.0001	1.72 (1.57, 1.87) <0.0001	1.72 (1.57, 1.88) <0.0001
ABSI Z score	1.11 (0.96, 1.26) <0.0001	0.44 (0.26, 0.61) <0.0001	0.44 (0.27, 0.62) <0.0001
BRI Z score	1.52 (1.37, 1.66) <0.0001	1.63 (1.48, 1.78) <0.0001	1.64 (1.48, 1.79) <0.0001

Data in the table: β (95%CI) *p*-value/OR (95%CI) *p*-value.

Outcome variables: Hypertension; Mean systolic blood pressure (mmHg); Mean diastolic blood pressure (mmHg).

Exposure variables: ABSI Z score; Waist (cm) Z score; BMI (kg/m^2^) Z score; WHtR Z score; BRI Z score.

Model I adjust for: None.

Model II, adjust for: Gender; Age (years); Race.

Model III, adjust for: Gender; Age (years); Race; Education level; Monthly family income; Avg alcohol drinks/day—past 12 months; Marital status; Activities time (Minutes)/day; Cigarettes/day during past 30 days.

After adjusting for all confounding factors, in the total sample, an increase of one standard deviation in WC, BMI, WHtR, ABSI and BRI, was associated with an increase in the occurrence of hypertension by 33% (95% CI: 27%–40%), 32% (95% CI: 26%–38%), 35% (95% CI: 28%–42%), 9% (95% CI: 4%–16%) and 32% (95% CI: 26%–38%), respectively.

Similarly, SBP increased by 2.36 mmHg (95% CI: 2.16–2.56), 2.41 mmHg (95% CI: 2.21–2.60), 2.48 mmHg (95% CI: 2.28–2.68), 0.42 mmHg (95% CI: 0.19–0.66) and 2.46 mmHg (95% CI: 2.26–2.66), respectively. DBP increased by 1.83 mmHg (95% CI: 1.68–1.98), 1.72 mmHg (95% CI: 1.58–1.87), 1.72 mmHg (95% CI: 1.57–1.88), 0.44 mmHg (95% CI: 0.27–0.62) and 1.64 mmHg (95% CI: 1.48–1.79), respectively.

### 3.6 ROC curve analysis

**FIGURE 2:**
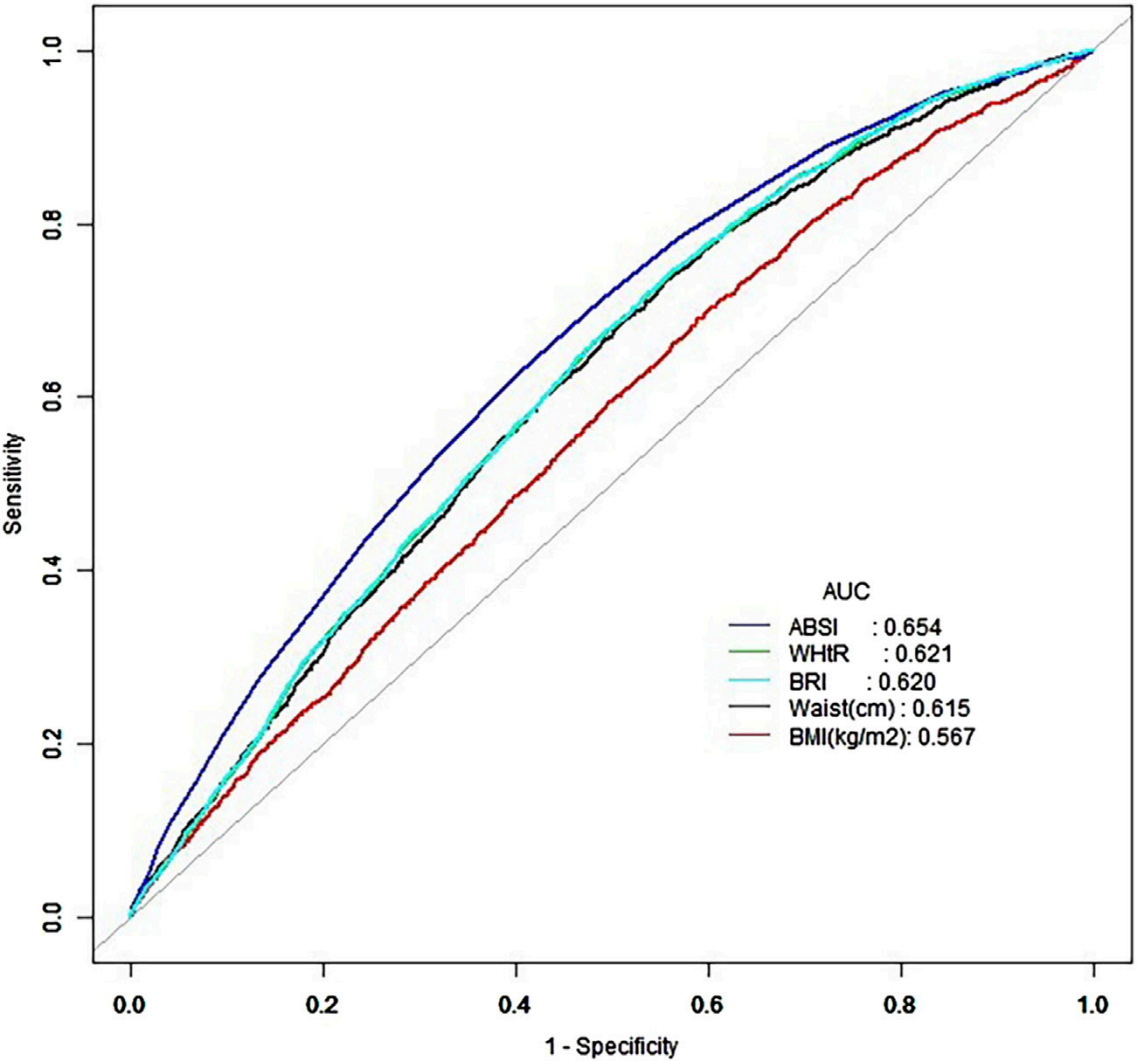
ROC curve of the overall population.

**FIGURE 3:**
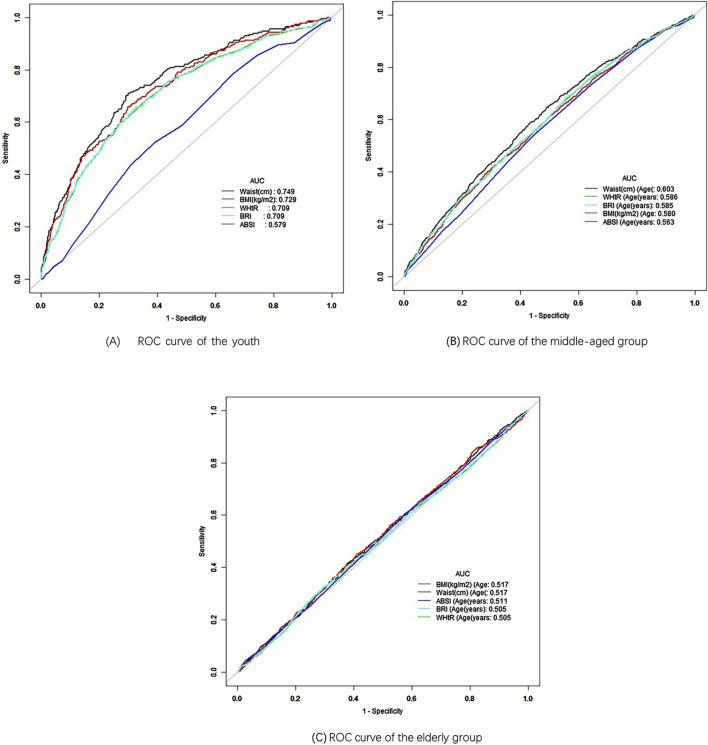
ROC curve of different age. **(A)** ROC curve of the youth. **(B)** ROC curve of the middle-aged group. **(C)** ROC curve of the elderly group.

ROC curve analysis was performed to assess the predictive performance of anthropometric indices in the overall population (Figure 2). The results indicated that all indices had moderate predictive performance, with AUC values below 0.7. Among these, ABSI had the highest AUC of 0.654, followed by WHtR, BRI and WC. In contrast, BMI exhibited the lowest AUC, indicating that ABSI had the best predictive ability in the overall population, while BMI showed the least. However, when stratified by age, the youth group (Figure 3A) exhibited better predictive performance, with ABSI having the lowest AUC at 0.579 (95% CI: 0.543–0.615). At the same time, the other four indices exceeded 0.7, indicating good predictive ability. Among these, WC performed best with an AUC of 0.749 (95% CI: 0.716–0.782) and a cutoff value of 98.25 cm. The BMI followed, with an AUC of 0.729 (95% CI: 0.696–0.763) and a cutoff value of 29.69 kg/m^2^. By coincidence, the WHtR and BRI had the same AUC of 0.709 (95% CI: 0.674–0.744), with cutoff values of 0.595 and 4.935, respectively.

In the middle-aged group (Figure 3B), WC again demonstrated the best performance with an AUC of 0.603 (95% CI: 0.585–0.621). However, the AUC values for the other four measurement indicators were all below 0.6, demonstrating their low predictive significance. Conversely, in the elderly group (Figure 3C), BMI had the highest AUC at 0.517 (95% CI: 0.497–0.537), indicating minimal predictive value. The AUC values for the other four predictive indicators were lower, indicating they had limited predictive ability. As age increased, the AUC values generally decreased, with predictive significance nearly disappearing in older people, consistent with the results of the age-stratified analysis (Table 6). These findings suggest that anthropometric indices have a stronger association with hypertension in younger individuals.

**FIGURE 4:**
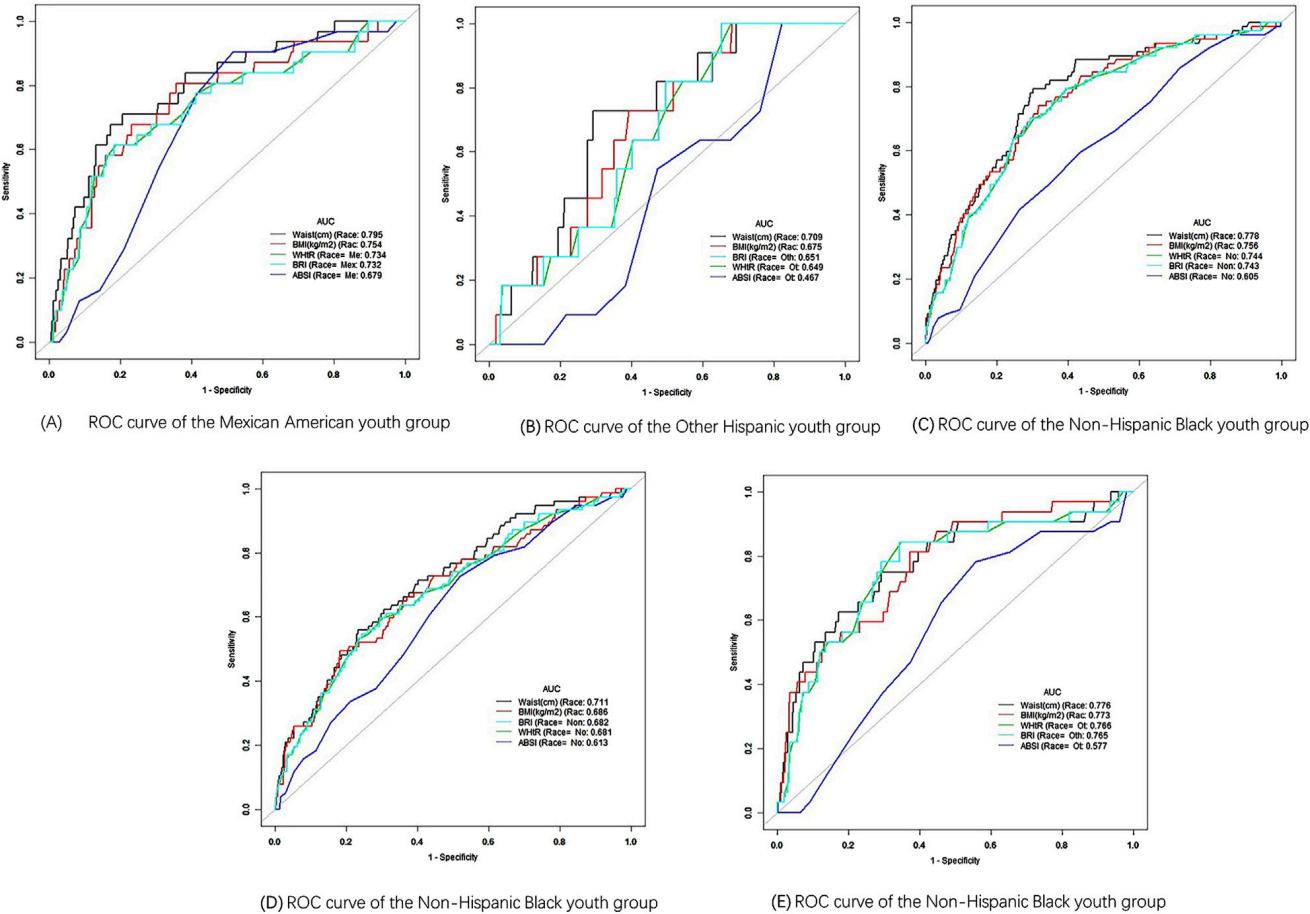
ROC curve of different race. **(A)** ROC curve of the Mexican American youth group. **(B)** ROC curve of the Other Hispanic youth group. **(C)** ROC curve of the Non-Hispanic Black youth group. **(D)** ROC curve of the Non-Hispanic Black youth group. **(E)** ROC curve of the Non-Hispanic Black youth group.

**FIGURE 5:**
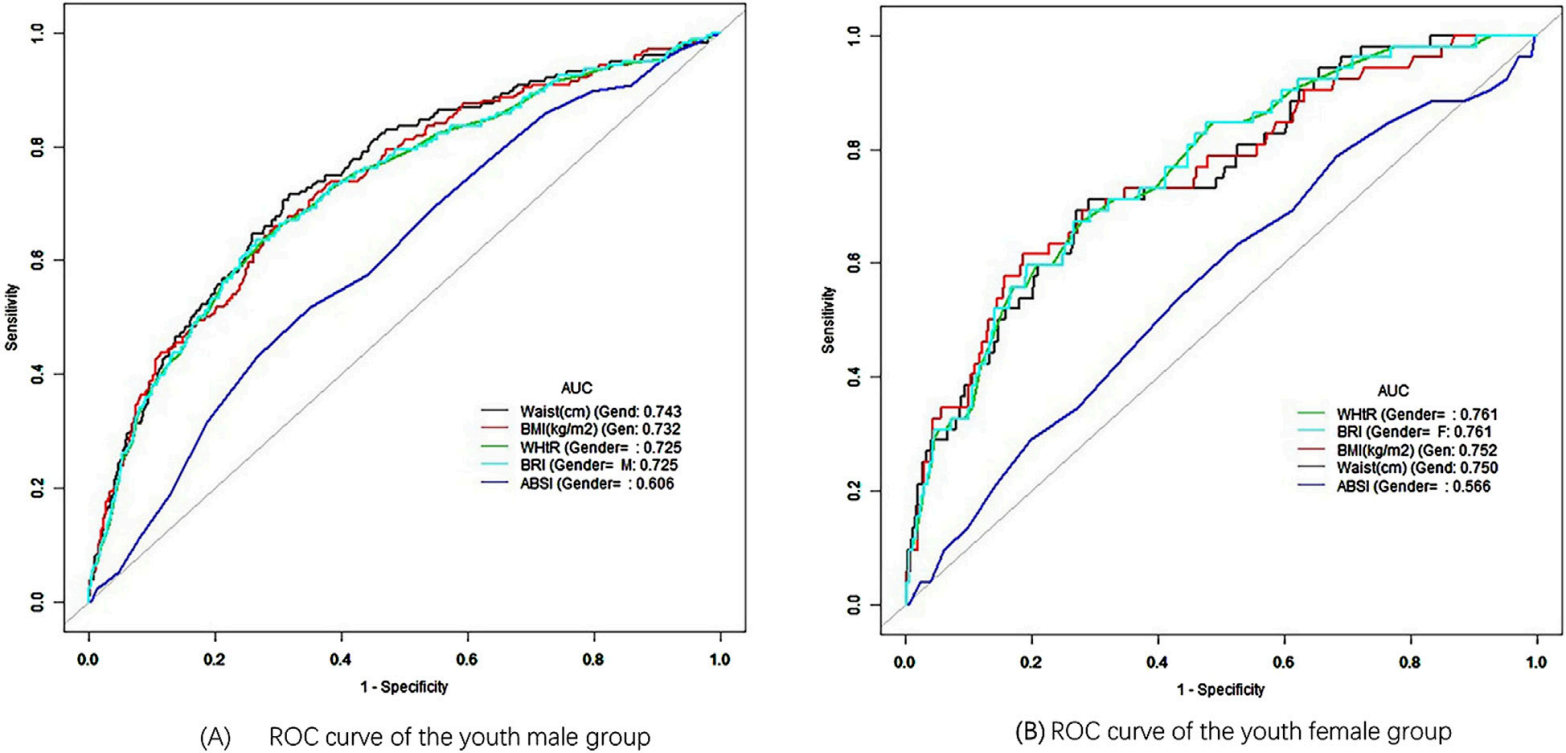
ROC curve of different gender. **(A)** ROC curve of the youth male group. **(B)** ROC curve of the youth female group.

Further stratification by race within the youth group revealed that WC had the highest AUC between different races, with WC achieving an AUC of 0.795 (95% CI: 0.714–0.877) for race 1 and a cutoff value of 108.75 cm, demonstrating excellent predictive performance (Figure 4A). The AUC areas for WC in other race groups were 0.709, 0.778, 0.711, and 0.776, ranking first overall and indicating strong predictive ability (Figures 4B–E). BMI ranked second in AUC area across all race groups, also demonstrating good predictive ability. In race 1, 3, and 5, WHtR ranked third in AUC area, while BRI ranked fourth (Figures 4A, C, E). In race 2 and 4, BRI ranked third in AUC, with WHtR in fourth place (Figures 4B, D). Additionally, the ABSI had the poorest performance among all race groups (Figure 4). In the gender-stratified analysis within the youth group, WC showed a significant AUC of 0.743 (95% CI: 0.704–0.782) for young males, with a cutoff value of 98.25 cm, While the AUC values for BMI, WHtR and BRI decreased in order, they all remain above 0.7, reflecting a good predictive ability (Figure 5A). Conversely, WHtR and BRI exhibited better predictive performance for young females, with AUC values of 0.761 (95% CI: 0.696–0.825) and cutoff values of 0.615 and 5.893, respectively, The AUC areas for BRI, BMI, and WC decreased sequentially, yet all remain above 0.7, indicating a good predictive ability (Figure 5B). After stratifying by gender, the AUC area for ABSI was the lowest, indicating weaker predictive ability (Figure 5). Bootstrap resampling analysis confirmed the stability of these results in the youth group (Supplementary Tables 1–6).

These findings suggest that, except for ABSI, the other anthropometric indices performed well in predicting hypertension within the youth population.

## 4 Discussion

This study examined the relationship between five non-invasive anthropometric indices, WC, BMI, WHtR, ABSI and BRI, and hypertension, using a large-scale data set from NHANES. Using multivariate regression analysis, we could quantify the specific impact of each index on hypertension occurrence, SBP and DBP. Our findings show that while ABSI had the smallest effect regarding β and OR values, BRI performed similarly to traditional measures such as WC, BMI and WHtR. This suggests that BRI may offer a valuable alternative for hypertension risk assessment. Still, the utility of ABSI as a predictive tool for hypertension is limited in this context (Krakauer and Krakauer, 2012).

The use of ROC curve analysis further elucidated the predictive power of these indices. In the youth group, WC demonstrated the highest predictive value for hypertension, with an AUC of 0.749. This finding highlights the importance of abdominal obesity as a significant risk factor for hypertension in younger populations, consistent with previous study that has emphasized the role of central adiposity in cardiovascular risk (Yusuf et al., 2005). However, as age increased, the predictive value of anthropometric indices generally decreased, particularly in the elderly group, where BMI exhibited an AUC of just 0.517. This reduction in predictive value may be due to the complex physiological changes that occur with aging, which could weaken the association between body fat distribution and hypertension (Franklin et al., 1997).

Our stratified analyses by gender and race revealed important nuances in how these indices perform in different demographic groups. For example, WC was the best predictor of hypertension in young males, while WHtR and BRI were more predictive in young females. This is consistent with the findings of previous study that has reported sex differences in body fat distribution and its association with cardiovascular risk factors (Lemieux et al., 1994). Additionally, the differences observed in AUC values between racial groups underscore the importance of considering ethnicity when evaluating the risk of hypertension using anthropometric indices. For instance, race 1 (Mexican American) exhibited the highest AUC for WC, indicating that WC may be a more sensitive predictor of hypertension in this population compared to others (Huxley et al., 2010).

ABSI, although a novel index designed to account for body shape independently of BMI, showed the weakest association with hypertension in almost all stratified analyses. This finding contrasts with some earlier study that has suggested that ABSI might offer an advantage in predicting mortality and other health outcomes, particularly in older adults (Krakauer and Krakauer, 2012). However, our results indicate that ABSI may not be as helpful for hypertension prediction, particularly in younger and middle-aged populations. However, the underlying mechanism remains unclear, and literature analysis indicates that it may be associated with differences in gender, age, and race (Chang et al., 2016; Ji et al., 2018; Tee et al., 2020). In this study, the predictive ability of ABSI was most effective in the overall population (Figure 2), while its performance decreased when stratified by age, race, and gender (Figures 3–5), reinforcing this conclusion. More research is warranted to explore whether ABSI has predictive value for other conditions beyond hypertension.

Our study also contributes to the ongoing debate about which anthropometric indices are most helpful in predicting hypertension. The similar performance of BRI compared to WC, BMI and WHtR suggests that BRI could serve as an alternative measure, particularly for clinicians seeking a single, non-invasive metric that captures both body roundness and abdominal obesity. However, the marginal differences in predictive value across these indices suggest that clinical decisions should not rely solely on one measure but consider a combination of anthropometric and clinical data for more accurate risk stratification (Ashwell et al., 2012).

Despite these significant findings, several limitations of our study must be acknowledged. First, the cross-sectional design of this study excludes any conclusions regarding causality between anthropometric indices and hypertension. Longitudinal studies are needed to establish whether changes in these indices over time can predict the development of hypertension and related cardiovascular events (Mann, 2003). Second, while the exclusion of individuals using antihypertensive medications ensured a cleaner data set focused on untreated blood pressure, it may have led to an underestimate of the true prevalence of hypertension, as many hypertensive individuals are under treatment (NCD Risk Factor Collaboration, 2021). Future research should explore the effects of antihypertensive treatment on the predictive power of anthropometric indices. Additionally, although our study used a large and representative dataset, it is important to consider potential confounding factors such as diet, physical activity and genetic predisposition, which were not fully accounted for in our analysis (Lamelas et al., 2019). Further studies should integrate these variables to better understand the complex relationships between anthropometric indices and hypertension risk.

## 5 Conclusion

This study provides valuable information on the predictive utility of non-invasive anthropometric indices for hypertension, particularly in younger populations. WC emerged as the strongest predictor, particularly for young males, while WHtR and BRI performed well for young females. ABSI, however, did not demonstrate superiority over traditional indices in this context. These findings underscore the importance of age, gender and race in interpreting anthropometric data for hypertension risk prediction. Future longitudinal studies are needed to validate these results and assess the long-term predictive value of these indices for hypertension prevention and management. Ultimately, these insights can help clinicians and pharmacists tailor treatment and prevention strategies based on simple, non-invasive measures of body composition.

## Data Availability

The original contributions presented in the study are included in the article/Supplementary Material, further inquiries can be directed to the corresponding authors.
