# Supplementary material for: Association between anthropometric indices and hypertension: identifying optimal cutoff points for U.S. adults across different populations
Source: Front Pharmacol. 2024 Dec 12;15:1503059. doi: 10.3389/fphar.2024.1503059 (PMC11669579; doi:10.3389/fphar.2024.1503059)
Supplement: Supplementary file 1 [file Table1.docx]

***Supplementary Material***

**Supplementary Tables**

**Supplementary Table 1.** ROC analysis for continuous predictor in the youth group

| Test | 1 | 0 | ROC area (AUC) | 95%CI low | 95%CI upp | Best threshold | Specificity | Sensitivity |
| --- | --- | --- | --- | --- | --- | --- | --- | --- |
| WC (cm) | 228 | 8016 | 0.7369 | 0.7077 | 0.7728 | 98.2500 | 0.6990 | 0.7105 |
| BMI (kg/m2) | 228 | 8016 | 0.7220 | 0.6857 | 0.7538 | 29.6900 | 0.7008 | 0.6579 |
| WHtR | 228 | 8016 | 0.6911 | 0.6518 | 0.7305 | 0.5950 | 0.7289 | 0.5965 |
| ABSI | 228 | 8016 | 0.5452 | 0.5081 | 0.5904 | 0.0795 | 0.6089 | 0.5219 |
| BRI | 228 | 8016 | 0.6996 | 0.6643 | 0.7358 | 4.9350 | 0.6753 | 0.6579 |

**Supplementary Table 2.** Best threshold analysis in the youth group

| Test | Best threshold | Specificity | Sensitivity | Accuracy | Positive-LR | Negative-LR | Diagnose-OR | N-for-diagnose | Postive-pv | Negative-pv | a | b | c | d |
| --- | --- | --- | --- | --- | --- | --- | --- | --- | --- | --- | --- | --- | --- | --- |
| WC (cm) | 98.2500 | 0.6990 | 0.7105 | 0.6993 | 2.3604 | 0.4141 | 5.6995 | 2.4420 | 0.0629 | 0.9884 | 162 | 2413 | 66 | 5603 |
| BMI (kg/m^2^) | 29.6900 | 0.7008 | 0.6579 | 0.6997 | 2.1992 | 0.4881 | 4.5054 | 2.7875 | 0.0589 | 0.9863 | 150 | 2398 | 78 | 5618 |
| WHtR | 0.5950 | 0.7289 | 0.5965 | 0.7253 | 2.2004 | 0.5536 | 3.9749 | 3.0731 | 0.0589 | 0.9845 | 136 | 2173 | 92 | 5843 |
| ABSI | 0.0795 | 0.6089 | 0.5219 | 0.6065 | 1.3345 | 0.7851 | 1.6998 | 7.6431 | 0.0366 | 0.9782 | 119 | 3135 | 109 | 4881 |
| BRI | 4.9350 | 0.6753 | 0.6579 | 0.6748 | 2.0260 | 0.5066 | 3.9991 | 3.0015 | 0.0545 | 0.9858 | 150 | 2603 | 78 | 5413 |

Positive pv: Positive Predictive Value; Negative pv: Negative Predictive Value Positive-LR: Positive Likelihood Ratio; Negative-LR: Negative Likelihood Ratio; Diagnosis OR: Diagnostic Odds Ratio.

The AUC confidence interval and significance test use a non-parametric bootstrap resampling method (Bootstrap resampling times = 500).

Outcome variable: Hypertension.

**Supplementary Table 3.** ROC analysis for continuous predictor in the youth group (Stratified analysis by gender)

| Test | 1 | 0 | ROC area (AUC) | 95%CI low | 95%CI upp | Best threshold | Specificity | Sensitivity |
| --- | --- | --- | --- | --- | --- | --- | --- | --- |
| WC (cm) (Gender= Male) | 176 | 4026 | 0.7185 | 0.6775 | 0.7626 | 98.2500 | 0.6823 | 0.7159 |
| BMI (kg/m2) (Gender= Male) | 176 | 4026 | 0.7194 | 0.6826 | 0.7577 | 29.3350 | 0.7059 | 0.6591 |
| WHtR (Gender= Male) | 176 | 4026 | 0.7054 | 0.6686 | 0.7466 | 0.5750 | 0.7228 | 0.6364 |
| ABSI (Gender= Male) | 176 | 4026 | 0.5834 | 0.5372 | 0.6329 | 0.0795 | 0.6495 | 0.5170 |
| BRI (Gender= Male) | 176 | 4026 | 0.7163 | 0.6785 | 0.7593 | 4.9350 | 0.7355 | 0.6364 |
| WC (cm) (Gender= Female) | 52 | 3990 | 0.7636 | 0.6900 | 0.8135 | 99.2500 | 0.7288 | 0.6923 |
| BMI (kg/m2) (Gender= Female) | 52 | 3990 | 0.7489 | 0.6789 | 0.8108 | 33.7750 | 0.8140 | 0.6154 |
| WHtR (Gender= Female) | 52 | 3990 | 0.7643 | 0.6944 | 0.8192 | 0.6150 | 0.7221 | 0.6731 |
| ABSI (Gender= Female) | 52 | 3990 | 0.5299 | 0.4565 | 0.6166 | 0.0785 | 0.4734 | 0.6346 |
| BRI (Gender= Female) | 52 | 3990 | 0.7617 | 0.7014 | 0.8174 | 5.8930 | 0.7338 | 0.6731 |

**Supplementary Table 4.** Best threshold analysis in the youth group (Stratified analysis by gender)

| Test | Best threshold | Specificity | Sensitivity | Accuracy | Positive-LR | Negative-LR | Diagnose-OR | N-for-diagnose | Postive-pv | Negative-pv | a | b | c | d |
| --- | --- | --- | --- | --- | --- | --- | --- | --- | --- | --- | --- | --- | --- | --- |
| WC (cm)  (Gender= Male) | 98.2500 | 0.6823 | 0.7159 | 0.6837 | 2.2535 | 0.4164 | 5.4124 | 2.5111 | 0.0897 | 0.9821 | 126 | 1279 | 50 | 2747 |
| BMI (kg/m2) (Gender= Male) | 29.3350 | 0.7059 | 0.6591 | 0.7040 | 2.2411 | 0.4829 | 4.6407 | 2.7397 | 0.0892 | 0.9793 | 116 | 1184 | 60 | 2842 |
| WHtR  (Gender= Male) | 0.5750 | 0.7228 | 0.6364 | 0.7192 | 2.2957 | 0.5031 | 4.5632 | 2.7842 | 0.0912 | 0.9785 | 112 | 1116 | 64 | 2910 |
| ABSI (Gender= Male) | 0.0795 | 0.6495 | 0.5170 | 0.6440 | 1.4753 | 0.7435 | 1.9841 | 6.0034 | 0.0606 | 0.9685 | 91 | 1411 | 85 | 2615 |
| BRI (Gender= Male) | 4.9350 | 0.7355 | 0.6364 | 0.7313 | 2.4056 | 0.4944 | 4.8655 | 2.6894 | 0.0952 | 0.9788 | 112 | 1065 | 64 | 2961 |
| WC (cm)  (Gender= Female) | 99.2500 | 0.7288 | 0.6923 | 0.7284 | 2.5530 | 0.4222 | 6.0471 | 2.3746 | 0.0322 | 0.9945 | 36 | 1082 | 16 | 2908 |
| BMI (kg/m2) (Gender= Female) | 33.7750 | 0.8140 | 0.6154 | 0.8115 | 3.3091 | 0.4725 | 7.0038 | 2.3287 | 0.0413 | 0.9939 | 32 | 742 | 20 | 3248 |
| WHtR  (Gender= Female) | 0.6150 | 0.7221 | 0.6731 | 0.7214 | 2.4216 | 0.4528 | 5.3485 | 2.5308 | 0.0306 | 0.9941 | 35 | 1109 | 17 | 2881 |
| ABSI  (Gender= Female) | 0.0785 | 0.4734 | 0.6346 | 0.4755 | 1.2052 | 0.7718 | 1.5616 | 9.2551 | 0.0155 | 0.9900 | 33 | 2101 | 19 | 1889 |
| BRI (Gender= Female) | 5.8930 | 0.7338 | 0.6731 | 0.7331 | 2.5288 | 0.4455 | 5.6763 | 2.4575 | 0.0319 | 0.9942 | 35 | 1062 | 17 | 2928 |

Positive pv: Positive Predictive Value; Negative pv: Negative Predictive Value Positive-LR: Positive Likelihood Ratio; Negative-LR: Negative Likelihood Ratio; Diagnosis OR: Diagnostic Odds Ratio.

The AUC confidence interval and significance test use a non-parametric bootstrap resampling method (Bootstrap resampling times = 500).

Outcome variable: Hypertension.

**Supplementary Table 5. ROC** analysis for continuous predictor in the youth group (Stratified analysis by race)

| Test | 1 | 0 | ROC area (AUC) | 95%CI low | 95%CI upp | Best threshold | Specificity | Sensitivity |
| --- | --- | --- | --- | --- | --- | --- | --- | --- |
| WC (cm) (Race= Mexican American) | 31 | 1444 | 0.7886 | 0.7045 | 0.8627 | 108.7500 | 0.8269 | 0.6774 |
| BMI (kg/m2) (Race= Mexican American) | 31 | 1444 | 0.7349 | 0.6503 | 0.8338 | 29.8300 | 0.6427 | 0.8065 |
| WHtR (Race= Mexican American) | 31 | 1444 | 0.7181 | 0.6370 | 0.7989 | 0.6550 | 0.8144 | 0.6129 |
| ABSI (Race= Mexican American) | 31 | 1444 | 0.6350 | 0.5556 | 0.7550 | 0.0795 | 0.4841 | 0.9032 |
| BRI (Race= Mexican American) | 31 | 1444 | 0.7203 | 0.6275 | 0.8138 | 6.7820 | 0.8179 | 0.6129 |
| WC (cm) (Race= Other Hispanic) | 11 | 870 | 0.7012 | 0.5633 | 0.8194 | 98.7500 | 0.7080 | 0.7273 |
| BMI (kg/m2) (Race= Other Hispanic) | 11 | 870 | 0.6703 | 0.5277 | 0.7951 | 28.3900 | 0.6069 | 0.7273 |
| WHtR (Race= Other Hispanic) | 11 | 870 | 0.6514 | 0.5048 | 0.7875 | 0.5050 | 0.3207 | 1.0000 |
| ABSI (Race= Other Hispanic) | 11 | 870 | 0.4511 | 0.3426 | 0.6143 | 0.0825 | 0.1782 | 1.0000 |
| BRI (Race= Other Hispanic) | 11 | 870 | 0.6460 | 0.5081 | 0.7563 | 3.5700 | 0.3471 | 1.0000 |
| WC (cm) (Race= Non-Hispanic White) | 77 | 2819 | 0.7702 | 0.7167 | 0.8260 | 98.7500 | 0.6974 | 0.7922 |
| BMI (kg/m2) (Race= Non-Hispanic White) | 77 | 2819 | 0.7451 | 0.6901 | 0.8010 | 28.6900 | 0.6829 | 0.7403 |
| WHtR (Race= Non-Hispanic White) | 77 | 2819 | 0.7313 | 0.6747 | 0.7840 | 0.5750 | 0.6953 | 0.7013 |
| ABSI (Race= Non-Hispanic White) | 77 | 2819 | 0.5741 | 0.5140 | 0.6583 | 0.0795 | 0.5644 | 0.5974 |
| BRI (Race= Non-Hispanic White) | 77 | 2819 | 0.7363 | 0.6816 | 0.7931 | 4.9335 | 0.7056 | 0.7013 |
| WC (cm) (Race= Non-Hispanic Black) | 77 | 1688 | 0.7112 | 0.6583 | 0.7701 | 104.0500 | 0.7660 | 0.5584 |
| BMI (kg/m2) (Race= Non-Hispanic Black) | 77 | 1688 | 0.6869 | 0.6267 | 0.7400 | 34.7500 | 0.8175 | 0.4935 |
| WHtR (Race= Non-Hispanic Black) | 77 | 1688 | 0.6762 | 0.6160 | 0.7377 | 0.5850 | 0.6985 | 0.5974 |
| ABSI (Race= Non-Hispanic Black) | 77 | 1688 | 0.6048 | 0.5375 | 0.6668 | 0.0755 | 0.4799 | 0.7273 |
| BRI (Race= Non-Hispanic Black) | 77 | 1688 | 0.6796 | 0.6163 | 0.7424 | 5.1695 | 0.7050 | 0.5974 |
| WC (cm) (Race= Other Race - Including Multi-Racial) | 32 | 1195 | 0.7607 | 0.6704 | 0.8553 | 91.9000 | 0.7071 | 0.7500 |
| BMI (kg/m2) (Race= Other Race - Including Multi-Racial) | 32 | 1195 | 0.7618 | 0.6681 | 0.8461 | 25.5150 | 0.6268 | 0.8125 |
| WHtR (Race= Other Race - Including Multi-Racial) | 32 | 1195 | 0.7320 | 0.6408 | 0.8369 | 0.5350 | 0.6519 | 0.8438 |
| ABSI (Race= Other Race - Including Multi-Racial) | 32 | 1195 | 0.5268 | 0.4463 | 0.6322 | 0.0775 | 0.4427 | 0.7812 |
| BRI (Race= Other Race - Including Multi-Racial) | 32 | 1195 | 0.7431 | 0.6596 | 0.8438 | 4.0520 | 0.6561 | 0.8438 |

**Supplementary Table 6.** Best threshold analysis in the youth group in the youth group (Stratified analysis by race)

| Test | Best threshold | Specificity | Sensitivity | Accuracy | Positive-LR | Negative-LR | Diagnose-OR | N-for-diagnose | Postive-pv | Negative-pv | a | b | c | d |
| --- | --- | --- | --- | --- | --- | --- | --- | --- | --- | --- | --- | --- | --- | --- |
| WC (cm) (Race= Mexican American) | 108.7500 | 0.8269 | 0.6774 | 0.8237 | 3.9128 | 0.3901 | 10.0296 | 1.9830 | 0.0775 | 0.9917 | 21 | 250 | 10 | 1194 |
| BMI (kg/m2) (Race= Mexican American) | 29.8300 | 0.6427 | 0.8065 | 0.6461 | 2.2568 | 0.3012 | 7.4935 | 2.2266 | 0.0462 | 0.9936 | 25 | 516 | 6 | 928 |
| WHtR (Race= Mexican American) | 0.6550 | 0.8144 | 0.6129 | 0.8102 | 3.3024 | 0.4753 | 6.9478 | 2.3402 | 0.0662 | 0.9899 | 19 | 268 | 12 | 1176 |
| ABSI (Race= Mexican American) | 0.0795 | 0.4841 | 0.9032 | 0.4929 | 1.7507 | 0.1999 | 8.7570 | 2.5820 | 0.0362 | 0.9957 | 28 | 745 | 3 | 699 |
| BRI (Race= Mexican American) | 6.7820 | 0.8179 | 0.6129 | 0.8136 | 3.3651 | 0.4733 | 7.1099 | 2.3214 | 0.0674 | 0.9899 | 19 | 263 | 12 | 1181 |
| WC (cm) (Race= Other Hispanic) | 98.7500 | 0.7080 | 0.7273 | 0.7083 | 2.4911 | 0.3852 | 6.4672 | 2.2972 | 0.0305 | 0.9952 | 8 | 254 | 3 | 616 |
| BMI (kg/m2) (Race= Other Hispanic) | 28.3900 | 0.6069 | 0.7273 | 0.6084 | 1.8501 | 0.4494 | 4.1170 | 2.9925 | 0.0229 | 0.9944 | 8 | 342 | 3 | 528 |
| WHtR (Race= Other Hispanic) | 0.5050 | 0.3207 | 1.0000 | 0.3292 | 1.4721 | 0.0000 | Inf | 3.1183 | 0.0183 | 1.0000 | 11 | 591 | 0 | 279 |
| ABSI (Race= Other Hispanic) | 0.0825 | 0.1782 | 1.0000 | 0.1884 | 1.2168 | 0.0000 | Inf | 5.6129 | 0.0152 | 1.0000 | 11 | 715 | 0 | 155 |
| BRI (Race= Other Hispanic) | 3.5700 | 0.3471 | 1.0000 | 0.3553 | 1.5317 | 0.0000 | Inf | 2.8808 | 0.0190 | 1.0000 | 11 | 568 | 0 | 302 |
| WC (cm) (Race= Non-Hispanic White) | 98.7500 | 0.6974 | 0.7922 | 0.6999 | 2.6181 | 0.2979 | 8.7871 | 2.0424 | 0.0667 | 0.9919 | 61 | 853 | 16 | 1966 |
| BMI (kg/m2) (Race= Non-Hispanic White) | 28.6900 | 0.6829 | 0.7403 | 0.6844 | 2.3342 | 0.3804 | 6.1367 | 2.3634 | 0.0599 | 0.9897 | 57 | 894 | 20 | 1925 |
| WHtR (Race= Non-Hispanic White) | 0.5750 | 0.6953 | 0.7013 | 0.6954 | 2.3015 | 0.4296 | 5.3571 | 2.5216 | 0.0591 | 0.9884 | 54 | 859 | 23 | 1960 |
| ABSI (Race= Non-Hispanic White) | 0.0795 | 0.5644 | 0.5974 | 0.5653 | 1.3714 | 0.7133 | 1.9225 | 6.1810 | 0.0361 | 0.9809 | 46 | 1228 | 31 | 1591 |
| BRI (Race= Non-Hispanic White) | 4.9335 | 0.7056 | 0.7013 | 0.7055 | 2.3819 | 0.4233 | 5.6263 | 2.4578 | 0.0611 | 0.9886 | 54 | 830 | 23 | 1989 |
| WC (cm) (Race= Non-Hispanic Black) | 104.0500 | 0.7660 | 0.5584 | 0.7569 | 2.3865 | 0.5765 | 4.1399 | 3.0823 | 0.0982 | 0.9744 | 43 | 395 | 34 | 1293 |
| BMI (kg/m2) (Race= Non-Hispanic Black) | 34.7500 | 0.8175 | 0.4935 | 0.8034 | 2.7047 | 0.6195 | 4.3656 | 3.2150 | 0.1098 | 0.9725 | 38 | 308 | 39 | 1380 |
| WHtR (Race= Non-Hispanic Black) | 0.5850 | 0.6985 | 0.5974 | 0.6941 | 1.9812 | 0.5764 | 3.4371 | 3.3800 | 0.0829 | 0.9744 | 46 | 509 | 31 | 1179 |
| ABSI (Race= Non-Hispanic Black) | 0.0755 | 0.4799 | 0.7273 | 0.4907 | 1.3982 | 0.5684 | 2.4601 | 4.8279 | 0.0600 | 0.9747 | 56 | 878 | 21 | 810 |
| BRI (Race= Non-Hispanic Black) | 5.1695 | 0.7050 | 0.5974 | 0.7003 | 2.0249 | 0.5711 | 3.5458 | 3.3071 | 0.0846 | 0.9746 | 46 | 498 | 31 | 1190 |
| WC (cm) (Race= Other Race - Including Multi-Racial) | 91.9000 | 0.7071 | 0.7500 | 0.7082 | 2.5607 | 0.3536 | 7.2429 | 2.1876 | 0.0642 | 0.9906 | 24 | 350 | 8 | 845 |
| BMI (kg/m2) (Race= Other Race - Including Multi-Racial) | 25.5150 | 0.6268 | 0.8125 | 0.6316 | 2.1770 | 0.2991 | 7.2773 | 2.2765 | 0.0551 | 0.9921 | 26 | 446 | 6 | 749 |
| WHtR (Race= Other Race - Including Multi-Racial) | 0.5350 | 0.6519 | 0.8438 | 0.6569 | 2.4238 | 0.2397 | 10.1120 | 2.0176 | 0.0609 | 0.9936 | 27 | 416 | 5 | 779 |
| ABSI (Race= Other Race - Including Multi-Racial) | 0.0775 | 0.4427 | 0.7812 | 0.4515 | 1.4018 | 0.4942 | 2.8368 | 4.4657 | 0.0362 | 0.9869 | 25 | 666 | 7 | 529 |
| BRI (Race= Other Race - Including Multi-Racial) | 4.0520 | 0.6561 | 0.8438 | 0.6610 | 2.4532 | 0.2382 | 10.3007 | 2.0007 | 0.0616 | 0.9937 | 27 | 411 | 5 | 784 |

Positive pv: Positive Predictive Value; Negative pv: Negative Predictive Value Positive-LR: Positive Likelihood Ratio; Negative-LR: Negative Likelihood Ratio; Diagnosis OR: Diagnostic Odds Ratio.

The AUC confidence interval and significance test use a non-parametric bootstrap resampling method (Bootstrap resampling times = 500).

Outcome variable: Hypertension.
